# Corrosion inhibition of mild steel in 1M HCl by D-glucose derivatives of dihydropyrido [2,3-d:6,5-d′] dipyrimidine-2, 4, 6, 8(1H,3H, 5H,7H)-tetraone

**DOI:** 10.1038/srep44432

**Published:** 2017-03-20

**Authors:** Chandrabhan Verma, M. A. Quraishi, K. Kluza, M. Makowska-Janusik, Lukman O. Olasunkanmi, Eno E. Ebenso

**Affiliations:** 1 Department of Chemistry, Indian Institute of Technology, Banaras Hindu University, Varanasi 221005, India; 2 Institute of Physics, Faculty of Mathematics and Natural Science, Jan Dlugosz University, Al. Armii Krajowej 13/15, 42-200 Czestochowa, Poland; 3 Material Science Innovation & Modelling (MaSIM) Research Focus Area, Faculty of Agriculture, Science and Technology, North-West University (Mafikeng Campus), Private Bag X2046, Mmabatho 2735, South Africa; 4 Department of Chemistry, Obafemi Awolowo University, Ile-Ife 220005, Nigeria

## Abstract

D-glucose derivatives of dihydropyrido-[2,3-d:6,5-d′]-dipyrimidine-2, 4, 6, 8(1H,3H, 5H,7H)-tetraone (GPHs) have been synthesized and investigated as corrosion inhibitors for mild steel in 1M HCl solution using gravimetric, electrochemical, surface, quantum chemical calculations and Monte Carlo simulations methods. The order of inhibition efficiencies is GPH-3 > GPH-2 > GPH-1. The results further showed that the inhibitor molecules with electron releasing (-OH, -OCH_3_) substituents exhibit higher efficiency than the parent molecule without any substituents. Polarization study suggests that the studied compounds are mixed-type but exhibited predominantly cathodic inhibitive effect. The adsorption of these compounds on mild steel surface obeyed the Langmuir adsorption isotherm. SEM, EDX and AFM analyses were used to confirm the inhibitive actions of the molecules on mild steel surface. Quantum chemical (QC) calculations and Monte Carlo (MC) simulations studies were undertaken to further corroborate the experimental results.

Mild steel is frequently used as a construction material in different industries due to its low cost and high mechanical strength. However, it is prone to corrosion in aqueous environment, especially acidic solution, which is usually involved in industrial exercises such as acid pickling, industrial acid cleaning, acid descaling and oil well acidizing processes^1^,^2^. Heterocyclic compounds have been reported as effective corrosion inhibitors because they can easily adsorb on metallic surface via their π- and non-bonding electrons, aromatic rings and polar functional groups, which act as adsorption centers^3^,^4^,^5^,^6^,^7^,^8^,^9^. Many of these heterocyclic compounds can be synthesized through various economically viable methods^3^.

Recently, green chemistry has attracted a great deal of attention because of the increasing needs to reduce environment pollution and to minimize side effects on human health^10^,^11^. Keeping this in mind, the current work in the field of corrosion inhibition is also directed towards the development of corrosion inhibitors using the principles of “green chemistry”. This includes the use multicomponent reactions (MCRs), which combines three or more reactants in one step. MCRs has proven to be a powerful tool in organic synthesis due to its operational simplicity, small number of steps, facile automation, and minimized waste generation. MCRs as a synthetic method also saves time and enhances atom economy^12^,^13^.

Carbohydrates are naturally occurring environmentally sustainable materials with several fascinating properties such as availability and high solubility in the polar solvents. This is due to the presence of a large number of hydroxyl (-OH) groups in their molecules^14^,^15^,^16^,^17^,^18^,^19^,^20^. Carbohydrate derivatives exhibit excellent therapeutic action against HIV infection, cancer, diabetes, etc. in addition to their antibiotics, anti-inflammatory, antimalarial, antiviral, and glycosylation inhibitors properties^21^,^22^,^23^. The chemicals derived from carbohydrates are also widely used in our day to day life as cosmetics, detergent, food, cloths, sweetening agent, lumber paper and so on^22^,^23^. Moreover, chemical transformations that involve using hexoses particularly glucose together with other biologically and industrially useful chemicals has become a top research topic in the recent time. This is because hexoses are comparatively more abundant and remains one of the primary renewable resources based natural feedstock chemicals^24^,^25^. Careful examination of the literature revealed that carbohydrates and their derivatives such as chitosan show excellent corrosion inhibiting properties^26^,^27^,^28^.

Carbohydrates and their derivatives have occupied central place in different fields of chemistry and their synthesis based on MCRs are considered to be one of the most versatile green and economic methods for synthesis of various heterocyclic compounds. In continuation of our works on corrosion inhibition property of carbohydrate based corrosion inhibitors^8^, the present study considers the synthesis and investigation of corrosion inhibition effect of three novel D-glucose derivatives of dihydropyrido [2,3-d:6,5-d′] dipyrimidine-2, 4, 6, 8(1H,3H, 5H,7H)-tetraone namely, 5-((1S,2R,3R,4R)-1,2,3,4,5-pentahydroxypentyl)-10-phenyl-9,10-dihydropyrido[2,3-d:6,5-d′]dipyrimidine-2,4,6,8 (1H,3H, 5H,7H)- tetraone (GPH-1), 10- (4-hydroxy phenyl)- 5-((1S, 2R, 3R, 4R) -1,2,3,4,5-pentahydroxypentyl)-9,10-dihydropyrido [2,3-d:6,5-d′] dipyrimidine-2, 4, 6, 8(1H,3H, 5H,7H)-tetraone (GPH-2) and 10- (4-(methoxyphenyl)-5-((1S, 2R, 3R, 4R)-1, 2, 3, 4, 5-pentahydroxypentyl)-9,10-dihydropyrido[2,3-d:6,5-d′]dipyrimidine-2, 4, 6, 8(1H, 3H, 5H, 7H)-tetraone (GPH-3). The synthesized compounds were tested as prospective inhibitors of mild steel corrosion in hydrochloric acid using gravimetric, electrochemical impedance spectroscopy (EIS), potentiodynamic polarization, scanning electron microscopy (SEM), energy dispersive X-ray spectroscopy (EDX), atomic force microscopy (AFM), quantum chemical calculations and Monte Carlo simulations techniques. To the best of our knowledge, these compounds have not been tested as corrosion inhibitors in any previous work.

It is worthy of mention that glucose based compounds were chosen as corrosion inhibitors in this study because, precursors such as glucose and aniline are readily available and relatively cheaper. The cost of glucose is ≈460 Rs./Kg as compared to the glucosamine (≈80,000 Rs./Kg) used in our earlier study^8^. Moreover, our previous study on glucosamine based compounds involved the use of benzaldehyde derivatives in place of aniline derivatives (used in the present study). Meanwhile, benzaldehyde and its derivatives are relatively more expensive than anilines as the estimated cost of benzaldehyde is 860 Rs./Lt, whereas the estimated cost of aniline is 800 Rs./Lt. The results of the weight loss, electrochemical, surface and computational analyses were in good agreement.

## Experimental Procedures

### Materials

#### Synthesis of D-glucose derivatives of dihydropyrido [2,3-d:6,5-d′] dipyrimidine-2, 4, 6, 8(1H,3H, 5H,7H)-tetraone (GPHs)

**Figure 1:**
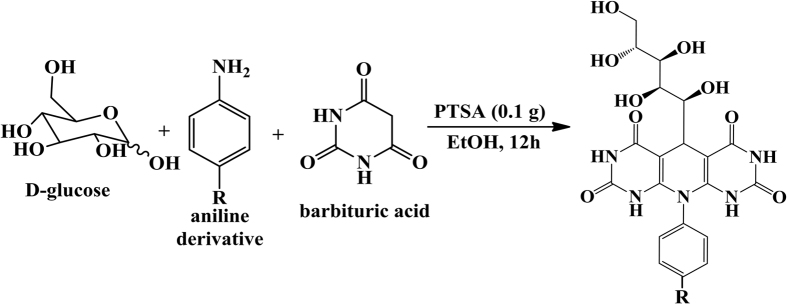
Synthetic scheme for the investigated inhibitors (GPHs).

The inhibitor molecules used in the present study were synthesized according to method described previously^29^. The experimental procedure involves stirring of 5 mL ethanolic solution of barbituric acid (2 mmol), glucose (1 mmol), aniline (1 mmol), and PTSA (0.1 g) at 50 °C for 24 h. The reaction mixture was cooled to room temperature after completion. Reaction completion was confirmed by TLC method using EtOAc-MeOH as eluent. The reaction mixture was filtered and crude product was washed three times with ethanol (3 × 5). The chemical structures, IUPAC names, abbreviations used for the synthesized D-glucose derivatives of dihydropyrido [2,3-d:6,5-d′] dipyrimidine-2, 4, 6, 8(1H,3H, 5H,7H)-tetraone (GPHs) are given in Table 1 and spectral data are given in Fig. S1 (Supplementary information). The synthetic scheme for the compounds is shown in Fig. 1.

### Electrodes and reagents

Mild steel specimens used as test materials contain carbon (C; 0.076%), manganese (Mn, 0.192%), phosphorus (P; 0.012%), silicon (Si; 0.026%), chromium (Cr; 0.050%), aluminum (Al; 0.023%), and the balance being iron (Fe). The mild steel specimens were polished with different grades of emery papers, washed with deionized water, degreased with acetone, dried under hot air blower and stored in desiccators. The gravimetric, electrochemical and surface measurements were carried out in the test solution of 1M HCl, which was prepared by dilution of 37% HCl (MERCK) with double deionized water. The volume of test solution for each measurement was 100 ml.

## Methods

### Weight loss measurements

The mild steel specimen used for the weight loss experiments was 2.5 × 2.0 × 0.025 cm^3^ dimension. The specimens were immersed in 100 ml of 1M HCl for 3 h in the absence and presence of several concentrations of the inhibitor molecules. The specimens were taken out after elapsed time and weighed accurately in order to determine the inhibition efficiency (*η*%) and surface coverage (*θ*) by employing following equations^30^,^31^:


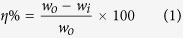



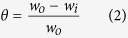


where, the weight losses of the mild steel specimens in the absence and presence of inhibitor molecules are denoted by *w*_0_ and *w*_i_, respectively.

### Electrochemical measurements

The electrochemical impedance spectroscopy (EIS) and potentiodynamic polarization measurements were carried out according to the procedure described elsewhere^30^,^31^. Similar to the weight loss experiments, the specimens were cleaned, washed, dried and stored in moisture free desiccators before starting the experiments. The typical procedure used for electrochemical measurements in the present study involved the combination of one sided 1 cm^2^ exposed surface area of the mild steel, 1 cm^2^ exposed area of pure platinum foil and saturated calomel electrode as working, counter and reference electrodes, respectively. These experiments were performed under potentiodynamic condition by employing the Gamry Potentiostat/Galvanostat (Model G-300) instrument. The electrochemical data were analyzed using Gamry Echem Analyst 5.0 software. The potentiodynamic polarization nature of mild steel in absence and presence of inhibitor molecules was investigated by varying the electrode potential from −0.25 to +0.25 V vs. corrosion potential (*E*_corr_) at a constant sweep rate of 1.0 mV s^−1^. Corrosion current density (*i*_corr_) was obtained by extrapolating the linear anodic and cathodic segments of the Tafel curves to the corrosion potential, and the inhibition efficiency (*η*%) was calculated using the values of *i*_corr_ according to the equation^30^,^31^:


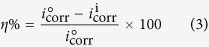


where, 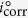 and 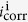 are corrosion current values in the absence and presence of inhibitor molecules respectively.

An AC signal of 10 mV peak-to-peak under frequency range of 100 kHz to 0.01 Hz at open circuit potential (OCP) was used to carry out electrochemical impedance measurements. The percentage inhibition efficiency (*η*%) of an inhibitor molecule was calculated from the values of charge transfer resistances (*R*_ct_) according to the equation^30^,^31^:


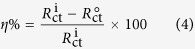


where, 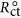 and 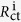 are charge transfer resistance values in the absence and presence of inhibitor molecules respectively.

### SEM measurements

The test material was cleaned, washed and dried similar to the procedure described in weight loss and electrochemical sections. The specimens were immersed in 100 mL test solution without and with optimum concentrations of the inhibitor molecules for 3 h. Thereafter, the specimens were taken out and their surface morphologies were assessed using SEM, EDX and AFM methods. At 500x magnification the surface morphology of the specimens by SEM method was evaluated using SEM model Ziess Evo 50 XVP instrument. The high energy dispersive X-ray spectroscopy (EDX) coupled with SEM was used to determine the elements present on the inhibited and uninhibited metallic surfaces. Whereas, surface morphology of the inhibited and uninhibited mild steel using AFM method was determined by employing NT-MDT multimode AFM, Russia, 111 controlled by solvers canning probe microscope controller instrument.

### Computational Methods

#### Quantum chemical calculations

The geometries GPH-1, GPH-2 and GPH-3 molecules were optimized to obtain the most stable ground state geometry based on lowest total energy. The initial geometry was obtained using ACD/Chem Sketch, an integrated software package from Advanced Chemistry Development, Inc. Then the structure was relaxed by applying the *ab initio* formalism implemented in GAMESS program package^32^,^33^. The minimum of the potential energy surface was calculated at restricted Hartree-Fock (RHF) level^34^ for neural molecules and at unrestricted Hartree-Fock (UHF) level for protonated ones with the 6-311G basis set in C_1_ symmetry. The quadratic approximation (QA) optimisation algorithm^35^ based on augmented Hessian techniques was used to reach the equilibrium geometry. The procedure was performed for molecules in vacuum as well as in water solution. In the latter case, the conductor-like PCM model (C-PCM)^36^,^37^,^38^ was used. The gradient convergence tolerance was chosen to be equal to 10^−4^ Hartree/Bohr. At the end of geometry search, the Hessian evaluation was performed to exclude the structures giving negative modes and ensure the thermodynamic equilibrium of the molecules.

The molecular structures with optimized geometries were used to calculate salient electronic properties. The electronic properties were computed for the neutral and protonated forms of the isolated molecules, and the neutral form in water phase. The calculations were carried out using the density functional theory (DFT) with B3LYP^39^,^40^,^41^ functional as implemented in GAMESS program package. The single point calculations were performed with 6-311G basis set augmented by polarisation and diffusion functions (6-311++G**)^42^,^43^,^44^. The RHF and UHF SCF energy convergence criterion was chosen to be equal to 10^−12^ Hartree.

#### Monte Carlo simulations

Adsorption of the GPH-1, GPH-2 and GPH-3 molecules at the (110) surface of Fe single crystal was investigated using Adsorption Locator module developed in BIOVIA Materials Studio programme package. The simulations were performed to find the preferential adsorption sites as well as adsorption energies of investigated molecules at the surface of the Fe crystal. The crystal structure of the Fe was build using builder module of Materials Studio. The Fe unit cell was formed in Im-3m space group with parameters a = b = c = 286.65 pm and α = β = γ = 90.00° 45. From the bulk material the (110) surface of Fe was specified. As the next step the adsorption of the GPH molecules at the Fe (110) surface was simulated in periodic boundary condition. To find the most stable adsorption sites series of total energy calculations using Adsorption Locator module were performed applying parameters as follow: the energy convergence criterion 10^−4^ kcal/mol, max force 0.005 kcal/mol/Å, max displacement = 0.005 Å utilizing Monte Carlo procedure with Dreiding force field^46^. The electrostatic interactions were calculated with Ewald method applying accuracy equal to 10^−4^ kcal/mol, charge group cut-off 15.5 Å and buffer width 0.5 Å. The van der Waals interactions were calculated using atom based summation method. The truncation was performed by cubic spline method with spline width equal to 1 Å and cut-off distance 15.5 Å.

## Results and Discussion

### Weight loss experiments

#### Effect of inhibitors concentration

Effect of concentrations on the inhibition efficiency of mild steel corrosion in 1M HCl is given in Table 2 and Fig. S2a (Supplementary information).

Inspection of the results show that inhibition performance of the studied compounds increases with increasing concentrations. It is attributed due to increase in the effective surface coverage (*η*%/100). The highest value of inhibition efficiencies, namely of 93.91%, 95.21% and 97.82% were obtained at 10.15 × 10^−5^ molL^−1^ concentration for GPH-1, GPH-2 and GPH-3, respectively. Further, enhancement in the concentrations did not cause any significant change in the inhibition efficiency indicating that 10.15 × 10^−5^ molL^−1^ is optimum concentration. The inhibition efficiency of the three studied molecules follows the order: GPH-3 > GPH-2 > GPH-1. This order of inhibition efficiency can be explained by the presence of the end-group attached to the phenyl ring of the aniline moiety. The higher inhibition performance of the GPH-2 and GPH-3 as compared to GPH-1 is attributed to presence of electron releasing hydroxyl (in GPH-2) and methoxy (in GPH-3) substituents at the 4^th^ position of the phenyl ring of the aniline moiety. These electron releasing groups in GPH-2 and GPH-3 increase the electron donating ability of inhibitor molecules toward the metallic surface by increasing their conjugations owing to presence of unshared electron pairs on the oxygen atoms and therefore enhances the inhibition performance^47^,^48^. Moreover, the higher inhibition efficiency of GPH-3 compared to GPH-2 might be attributed to presence of methoxy (-OCH_3_) group at 4^th^ position of the phenyl ring of aniline moiety in GPH-3 which has higher electron releasing tendency as compared to the hydroxyl (-OH) group present at 4^th^ position of the phenyl ring of aniline moiety in GPH-2^31^,^47^,^48^,^49^.

#### Effect of temperature

The weight loss experiments was also performed at different temperature ranging from 308 to 338 K in order to study the effect of temperature on the inhibition performance of the GPH inhibitor molecules and also to derived selected kinetic as well as thermodynamic parameters. The results are depicted in the Table 3 and Fig. S2b (Supplementary information) indicating that inhibition performance of the GPH molecules decreases with increasing solution temperatures.

**Figure 2:**
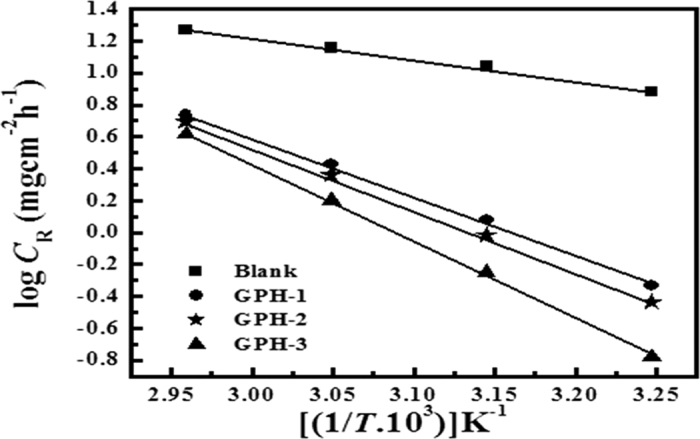
Arrhenius plots for the corrosion of mild steel in 1* *M HCl without and with the inhibitors.

The decreased inhibition efficiency on elevating the solution temperature might be due to increase in the mobility of the inhibitor molecules which in turn decreases the interaction between metallic surface and inhibitor molecules^50^,^51^. Moreover, rapid etching, molecular rearrangement and/or fragmentation and desorption of the adsorbed inhibitor molecules at elevated temperature may also decrease the inhibition efficiency^47^,^48^,^49^. The Arrhenius equation can be used successfully to explain the effect of temperature on the inhibition performance of studied compounds. It is represented by following equation^30^,^47^,^48^,^49^:


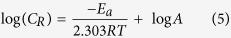


where, *C*_R_ represents the corrosion rate in mg cm^−2^ h^−1^, *A* represents the Arrhenius pre-exponential factor, *E*_*a*_ denotes the apparent activation energy, *R* is the universal gas constant and *T* represents the absolute temperature. The values of apparent activation energy in the absence and presence of inhibitor molecules was calculated from the slope (−∆*E*_*a*_/2.303*R*) values of Arrhenius plots represented in Fig. 2. The values of *E*_a_ were 70.55, 74.04 and 92.22 kJmol^−1^ for GPH-1, GPH-2 and GPH-3, respectively. It can be seen from the results that values of *E*_a_ in presence of inhibitor molecules were much higher than the blank (28.48 kJmol^−1^) which clearly indicates that in presence of inhibitor molecules more energy barrier have been achieved for metallic corrosion owing to the formation of inhibitors protective film, which eventually decreases the corrosion rate^52^.

#### Adsorption isotherm

The adsorption of the inhibitor molecules on the solid metallic surfaces is an important process that is associated with their inhibition efficiency. Depending upon the nature of adsorbent and adsorbate, the adsorption may be physical or chemical. Several adsorption isotherms were suggested to describe the adsorption process. In our present case, among various tested isotherm, the Langmuir isotherm gave the best fit. The best adsorption isotherm in the present study was chosen based on the value of regression coefficient (*R*^2^) for each tested isotherm. The values of *R*^2^ were most close to one for Langmuir adsorption isotherm. The value of regression coefficient slopes and intercept derived for Temkin, Freundlich and Langmuir adsorption isotherms are given in Table S1 (Supplementary Information). From the results it can be seen that although the values of *R*^2^ were more close to unity for Langmuir adsorption isotherm compared to Temkin and Freundlich adsorption isotherms, however the Langmuir isotherm is not strictly followed as the values of slopes are significantly deviated from unity (Table S1). The intermolecular interaction between adsorbed inhibitor molecules, which is, not considered during formulation of Langmuir adsorption isotherm equation, may be one of the reasons for this deviation^53^,^54^. The Langmuir isotherm is based on the hypothesis that there exists an equivalent number of active sites where only one molecule of adsorbate may be adsorbed to the metallic surface of the adsorbent. The Langmuir isotherm can be represented as follows^48^,^49^:


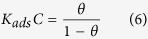


**Figure 3:**
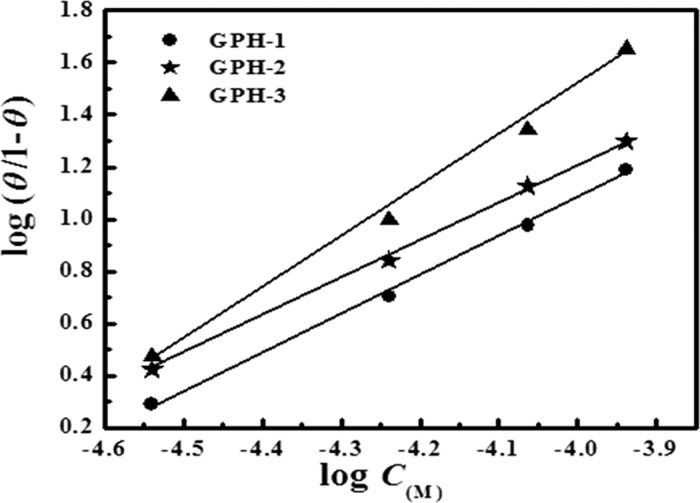
Langmuir adsorption isotherm plots for the adsorption of GPHs on mild steel surface in 1 M HCl at 308 K temperature.

In the above equation, *K*_ads_ represents the adsorption-desorption constant, *C* represents the concentration of the inhibitor, *θ* is the surface coverage (*θ* = *η%/100*). The values of *K*_ads_ for studied inhibitor molecules at different temperature were obtained with help of Langmuir isotherm plots shown in Fig. 3. The Temkin and Frumkin isotherms are shown in Fig. S3 (Supplementary information). The value of standard free energy of adsorption (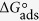) depends upon value of *K*_ads_ according to the following relation:


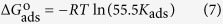


In the above relationship the numerical value 55.5 represents the water concentration in acidic solution. The values of (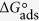) and *K*ads are represented in Table 4.

The value of (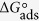) gives information about interaction between inhibitor molecules and metallic surface. Generally, the value of (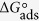) −20 kJmol^−1^ or less negative is associated with electrostatic interaction (physisorption) between oppositively charged inhibitors and metallic surface^55^,^56^, while the value of (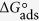) −40 kJmol^−1^ or more negative is connected with sharing of charges (chemisorption) between inhibitors and metallic surface^55^,^57^,^58^. From the results depicted in Table 4 it can be seen that values of (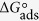) in our present measurement range from 33.72 kJmol^−1^ to 38.19 kJmol^−1^ indicating that GPH molecules interacted with the mild steel surface by both physisorption and chemisorption modes^47^,^48^,^49^,^55^,^57^,^58^,^59^,^60^.

### Electrochemical measurements

#### Polarization studies

**Figure 4:**
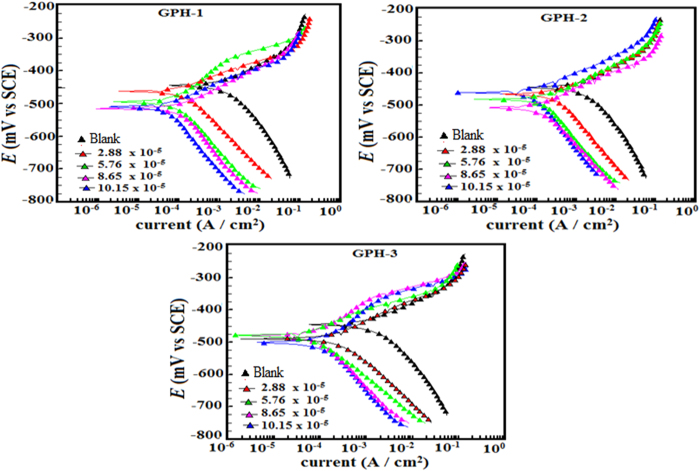
Potentiodynamic polarization curves for mild in the absence and presence of optimum concentrations of the studied inhibitors (GPHs).

The anodic and cathodic polarization curves for inhibited and uninhibited mild steel specimens were recorded in order to determine the electrochemical nature of studied inhibitor molecules. These polarization curves are shown in Fig. 4. For the inhibited and uninhibited metallic specimens, the values of Tafel polarization parameters including the anodic and cathodic Tafel slopes (*β*_a_ and *β*_c_ respectively) and corrosion current density (*i*_corr_) were obtained by extrapolating the linear segments of the anodic and cathodic Tafel curves to the corrosion potential (*E*_corr_). The percentages of inhibition efficiency along with the evaluated parameters are given in Table 5. From the results shown in Fig. 4 and Table 5 it can be seen that presence of inhibitors exerted significant effect on both anodic and cathodic reactions indicating that studied inhibitors retarded the both anodic metallic dissolution and cathodic hydrogen evolution. Further, from Table 5 it can also be seen that presence of inhibitors decreases the value of corrosion current density (*i*_corr_); the maximum decrease in *i*_corr_ value was observed at 10.15 × 10^−5^ mol/L concentration. The decreased value of *i*_corr_ in presence of inhibitors particularly at higher inhibitors concentrations is mainly attributed due to adsorption on inhibitor molecules over the metallic surface^61^. Whether, an inhibitor is anodic, cathodic or mixed type is determined by the displacement in the value of *E*_corr_, if the displacement in the value of *E*_corr_ for inhibited specimen is more than 85 mV as compared to the *E*_corr_ value of uninhibited specimen then the inhibitor can be categorized as anodic or cathodic type. However, if the displacement in the *E*_corr_ value is less than 85 mV then the inhibitor can be categorized as mixed type^61^,^62^,^63^. In our present study, the values of *E*_corr_ show irregular trends. The results depicted in Table 5 showed that values of *β*_c_ are more affected as compare to the values of *β*_a_ indicating the studied inhibitors act as cathodic type inhibitors^47^,^48^,^49^,^61^,^62^,^63^.

#### Electrochemical impedance spectroscopy studies

**Figure 5:**
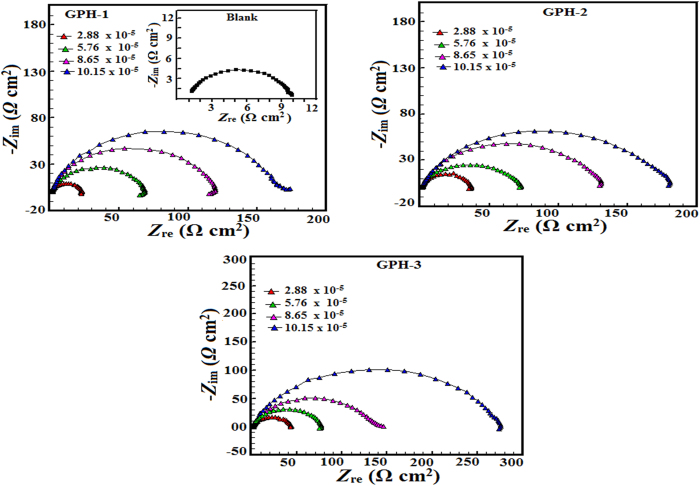
Nyquist plot for mild steel in 1 M HCl in the absence and presence of optimum of concentrations of GPHs.

The EIS is an important technique to monitor *in situ* electrochemical changes and also to develop understanding of the physical processes taking place at the metal/electrolyte interface^47^,^49^,^62^. Typical Nyquist plots for mild steel corrosion in the absence and presence of different concentrations of the studied inhibitor molecules is shown in Fig. 5. The impedance spectra for inhibited and uninhibited specimens give signal capacitive loop which indicates that studied inhibitor molecules behave as interface inhibitors i.e. they inhibit corrosion by adsorbing at metal/electrolyte interface^63^,^64^. The EIS parameters were obtained by analyzing the experimental EIS spectral with the aid of suitable equivalent circuit. The adopted equivalent circuit consists of *R*_s_ (solution resistance of the electrolyte), in series with parallel arrangement of *R*_ct_ (charge transfer resistance) and constant phase element (CPE) as contained in literature^64^,^65^. Charge transfer from the anode to the cathode, which results in oxidation of the metal is often somewhat restricted by the solvent molecules of the aqueous acid solution. This resistance by the electrolyte solution is referred to as the solution resistance (*R*_s_). The charge transfer resistance (*R*_ct_) refers to the ability of the protective film of organic molecules adsorbed on metallic surface to impede the transfer of charges across the metal/solution interface. For better approximation of impedance data, the capacitance was replaced by constant phase element (CPE)^48^,^49^,^65^. The CPE is defined in impedance representation as:


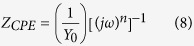


where, *Y*_0_ represents the CPE constant, *ω* is the angular frequency; *j* represents the imaginary number (i.e. *i*^2^ = −1) and *n* is the exponent (phase shift) which measure of surface inhomogeneity.

Generally, higher value of n is associated with lower surface roughness and vice versa. Moreover, the nature of CPE can also be explained based on the values of phase shift (n) and the CPE constant (Y0) for example *n* = 0 and *Y*0 = *R* represents the resistance, *n* = 1 and *Y*0 = *C* represents the capacitance, *n* = −1 and *Y*0 = 1/*L* represents the inductance, and *n* = 1/2 and *Y*0 = *W* represents the Warburg impedance (*Z*w)^8^,^47^,^48^,^49^. The following equation was used to calculate the double layer capacitance (Cdl)^66^:


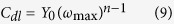


where, *ω*_max_ represents the frequency at which the imaginary quantity of impedance has gained the maximum (rad s^−1^) value. The parameters derived from electrochemical impedance data are presented in Table 6. Careful examination of the results revealed the significant increase in the value of *R*_ct_ in presence of inhibitor molecules which is attributed due to adsorption of inhibitor molecules on the metallic surface^56^,^67^. Further, decreased value of *C*_dl_ in presence of inhibitor molecules is attributed due to decrease in dielectric constant and/or increase in the thickness of electric double layer^56^,^67^. Moreover, closeness of *n* values to the unity clearly suggests that electric double layer form over the metallic surface is of capacitive nature.

**Figure 6:**
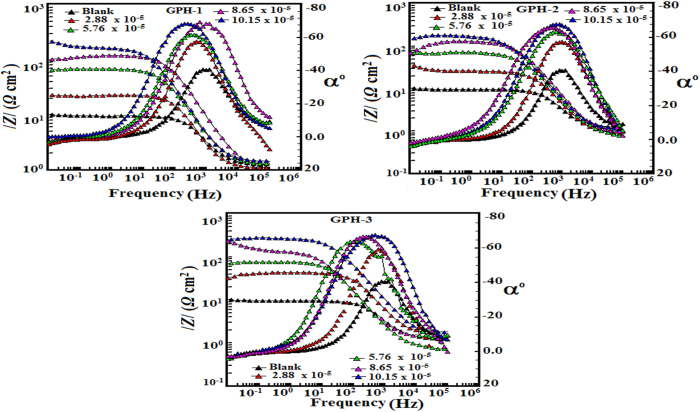
Bode plots for mild steel in 1 M HCl in the absence and presence of optimum of concentrations of GPHs.

In order to further study the electrochemical nature of studied inhibitor molecules, the Bode impedance and phase angle plots for inhibited and uninhibited metallic specimens were recorded and given in Fig. 6. The ideal capacitive behavior which is characterized by value of slope as unity and phase angle −90° 47,49, however, the deviation occurs in our present case as the values of slope and phase angle nearly were 0.85 and −70°, respectively. Values of slope, intercept, and regression coefficient and phase angle are given in Table S2. Deviation from the ideal capacitive behavior of the electric double layer in present case could be possible as result of surface inhomogeneity. The corrosion of metallic surface also resulted into the surface inhomogeneity. The Bode plots depicted in Fig. 6 it can be seen that values of phase angle for inhibited specimens are much higher than the uninhibited specimen. The increased values of phase angle for inhibited specimens indicated that surface becomes appreciably smooth owing to the formation of protective film by inhibitors over the metallic surface^47^,^49^,^56^. The broadening of the single maximum in the Bode plots further supports the protective film formation by inhibitor molecules^47^,^49^,^56^.

### Surface measurements

#### SEM and EDX analyses

**Figure 7:**
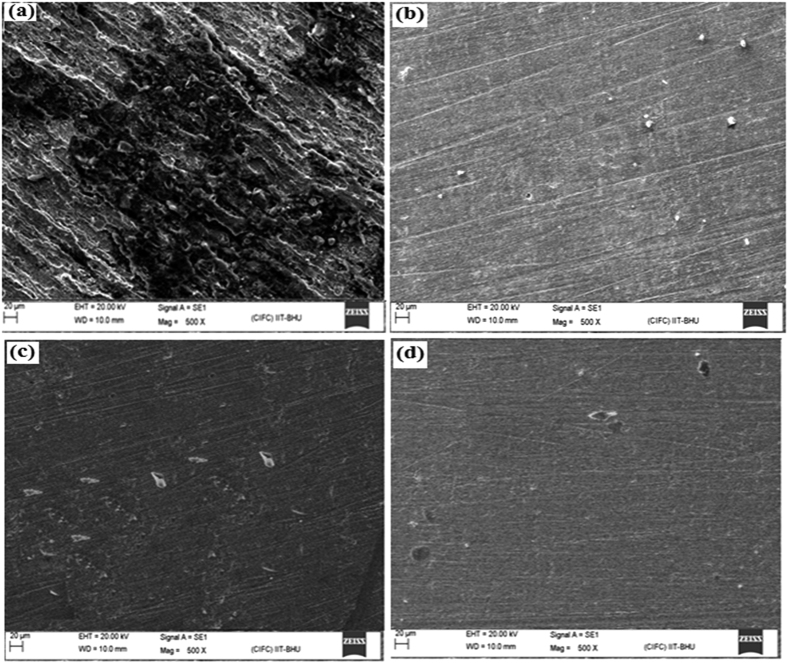
SEM images of mild steel surfaces: in 1 M HCl without GPHs (**a**), in 1 M HCl in the presence of optimum concentration of GPH-1 (**b**), GPH-2 (**c**), and GPH-3 (**d**).

Scanning electron microscopy (SEM) along with energy dispersive X-ray (EDX) spectroscopy is most widely used and best known surface analytical method for surface characterization. Generally, SEM analysis provides high resolution images of the surface and EDX analysis provides elemental identification in addition to quantitative compositional information. The SEM micrographs of inhibited and uninhibited metallic surface are given in Fig. 7 and their corresponding EDX spectra are given in Fig. S4 (Supplementary Information). The SEM micrograph of uninhibited mild steel surface showed highly corroded and damaged area along with pits and cracks which is attributed due to acid attack of metal. However, SEM images of the inhibited metallic surface are comparatively smoothed. The smoothed surface of the mild steel specimens in presence of inhibitor molecules is attributed due to their adsorption on the metallic surface. Moreover, film forming tendency of the inhibitor molecules was supported by EDX spectra of the inhibited and uninhibited mild steel surface (Fig. S4). Inspection of the EDX spectrum for uninhibited specimen shows characteristic signals for carbon, iron and oxygen. The presence of signal corresponding to oxygen in the EDX spectrum is might be attributed due to formation of oxide layer during the SEM/EDX operation. However, the EDX spectra for inhibited specimens showed additional signal for nitrogen which suggests the presence of inhibitor molecules over the mild steel surface. Moreover, increased intensity of signal corresponding for oxygen for inhibited specimens also indicated the film formation ability of these molecules.

#### Atomic force microscopy (AFM)

**Figure 8:**
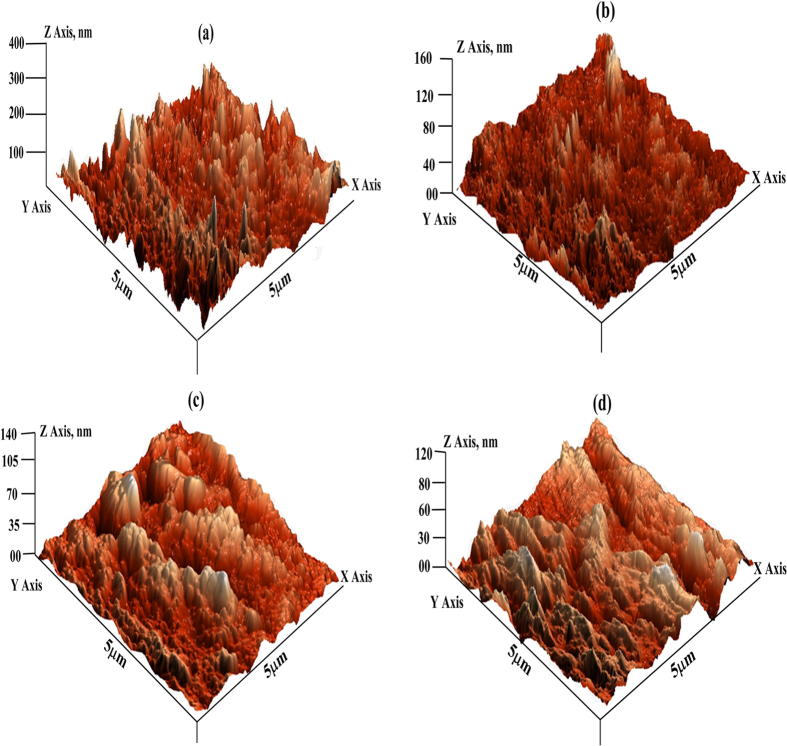
AFM images of mild steel: (**a**) in 1 M HCl in the absence of GPHs, and in the presence of optimum concentration of (**b**) GPH-1, (**c**) GPHB-2, and (**d**) GPH-3.

Nowadays, AFM has become a powerful technique and new choice to study the influence of inhibitor on the corrosion of metal in various electrolytic media. The AFM micrographs of inhibited and uninhibited metallic specimens are given in Fig. 8. Careful examination of surface morphology of the uninhibited mild steel specimen (Fig. 8a) revealed that the metallic surface is highly corroded and damaged. This finding suggests that the metallic surface is highly corroded due to free acid corrosion in uninhibited solution.

The calculated average surface roughness of uninhibited mild steel specimen was 390 nm. However, the AFM micrographs of inhibited specimens (Fig. 8b–d) show remarkable smoothness in the surface morphology. The calculated average surface roughness’s were 154 nm, 138 nm and 116 nm for inhibited mild steel specimens for GPH-1, GPH-2 and GPH-3, respectively. This significant improvement in the surface morphology of the inhibited mild steel specimens revealed that studied inhibitor molecules form protective surface covering that isolates the metal from electrolyte and save from corrosion.

### Computational studies

#### Quantum chemical calculations

**Figure 9:**
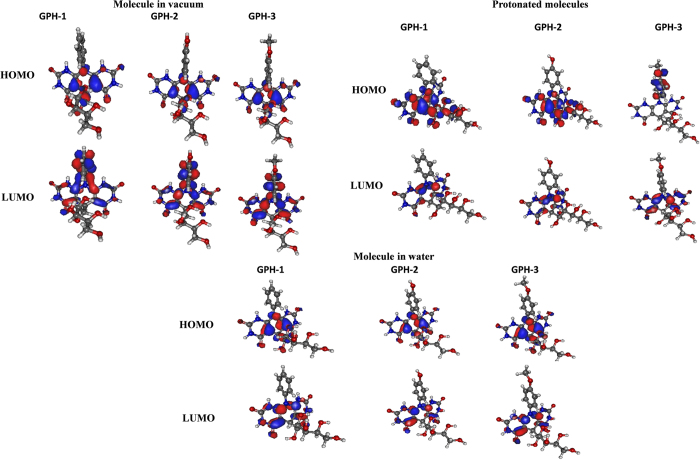
Shape of the HOMO and LUMO orbitals calculated for neutral and protonated molecules in vacuum and neutral molecules in water using DFT/B3LYP methodology.

The investigated molecules GPH-1, GPH-2 and GPH-3 differ from one to another by the end group attached to the phenyl ring of the aniline moiety. In consequence of geometry optimization procedure one may conclude that the skeleton of the investigated molecules is planar and the phenyl group is twisted depending on the supplementing hydroxyl or methoxy group. The substituents also change the electronic properties of the molecules. The electronic properties of the GPHs were calculated applying DFT/B3LYP potential with 6−311++G** basis set. The highest occupied molecular orbital (HOMO) and the lowest unoccupied molecular orbital (LUMO) studied for inhibitor molecules in vacuum, water and protonated forms are shown in Fig. 9. The calculated parameters such as energy of frontier molecular electrons belonging to HOMO and LUMO level *E*_HOMO_ and *E*_LUMO_, respectively, the Δ*E* = *E*_LUMO_ − *E*_HOMO_, dipole moment (*μ*) and electronegativity (*χ*) are presented in Table 7. The value of *E*_HOMO_ energy which indicates electron donating ability of the inhibitor molecules, in our present case obeyed the order: GPH-3 > GPH-2 > GPH-1. It clearly indicates that GPH-3 molecule has strongest electron donating tendency while for the GPH-1 mentioned parameter is the smallest^68^,^69^. The trend of values of *E*_HOMO_ observed for studied inhibitor molecules well supports their experimentally measured inhibition efficiency. The electron releasing groups (hydroxyl and methoxy) in GPH-2 and GPH-3 increase the electron donating ability of inhibitors as it was shown in paragraph 3.1.1.

In contrast to *E*_HOMO_, value of *E*_LUMO_ is a measure of electron affinity of the inhibitor molecule^68^,^69^. In our present case, values of *E*_LUMO_ did not show any regular trends. Moreover, Δ*E* is another important parameter which describes the interaction between inhibitor molecule and metallic surface. Generally, an inhibitor with lower value Δ*E* is consisted with high chemical reactivity and therefore associated with higher inhibition performance as compare to an inhibitor with high value of Δ*E*. The investigated inhibitor molecules GPH-1, GPH-2 and GPH-3 have almost the same electronic energy gap (Δ*E*) in the range of 4.5 eV. The relative electron donating ability of the studied inhibitor molecules can also be described with the help of their electronegativity (*χ*). In general, an inhibitor molecule with lower electronegativity (*χ*) is associated with higher electron donating tendency and therefore exhibited higher inhibition efficiency as compare to an inhibitor with higher value of electronegativity. In our present case values of electronegativity follows the trends: GPH-1 > GPH-2 > GPH-3, which converse with the order of inhibition efficiency and well support the experimental trend. The dipole moment is an important parameter which can be used in order to correlate relative interaction of inhibitor molecules with the metallic surface. Generally, an inhibitor with higher value of dipole moment is consisted with higher polarizability and higher effective surface area and therefore would be better corrosion inhibitor as compare to the inhibitor molecule with lower value of dipole moment. In the present case, the value of dipole moment of studied inhibitor molecules follows the order: GPH-3 > GPH-2 > GPH-1, which implies that GPH-3 has maximum effective surface area and would be best inhibitor among the studied compounds. Moreover, from Table 7 it can be seen that the values of dipole moment for studied inhibitor molecules are higher as compare to the value of dipole moment of water (1.85 Debye) indicating that these inhibitors have greater tendency to interact with metal as compare to the water. On this basis it can be conclude that these inhibitor molecules can adsorb on the mild steel surface by replacing the previously adsorbed water molecules^70^,^71^,^72^. Almost similar trends of the quantum chemical calculations indices were obtained for studied inhibitor molecules for aqueous and protonated molecules. From the results depicted in Table 7 it can be seen that the protonated inhibitor molecules have higher electronegativity while water environment decreases electronegativity. From the results, it can also be observed that for all non-protonated systems the HOMO orbital are created by the same active centres’. The HOMO orbitals lie at the skeleton of the molecules. The LUMO orbitals are shifted to the phenyl ring for all three studied molecules with and without electron releasing hydroxyl and methoxy groups. For the protonated molecules GPH-1 and GPH-2 the HOMO orbital is created by the electrons from molecule skeleton and for the GPH-3 at the phenyl ring. The LUMO orbitals of the protonated molecules are located at the molecule skeleton for all studied inhibitor molecules. The water environment does not change the location of HOMO orbitals compare to non-protonated molecule in vacuum but the LUMO looks like for the protonated molecules. It is due to the electrostatic interaction between inhibitors and environment and the intra-molecular charge transfer caused by the water interaction.

### Monte Carlo simulations

**Figure 10:**
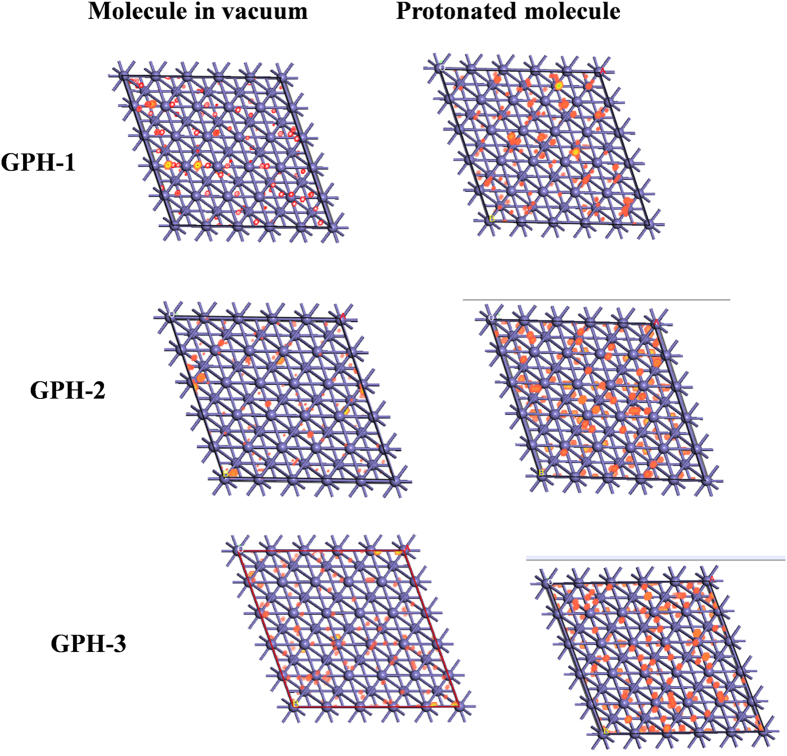
Top views of the density distribution of the most stable low energy configuration for the adsorption of inhibitor molecules at Fe (110) surface obtained by Monte Carlo simulations.

In order to understand the interaction between the inhibitor molecules and metal surface the Monte Carlo simulations (MC) were performed. The obtained results help to predict the most stable adsorption sites of the GPH molecules on metal surface. The top views of the density distribution of the most stable low energy configuration for the adsorption of inhibitor molecules at Fe (110) surface obtained by Monte Carlo simulations is shown in Fig. 10. The calculated data obtained by the MC simulation are presented in Table 8. The adsorption energy of the neutral molecules reproduces very well the experimental results. The large negative sign of adsorption energy (*E*_ads_) for all studied inhibitor molecules suggested that they have strong tendency of adsorption^73^,^74^. The water environment encourages absorption of molecules on the metal surface. In the Fig. 10 the density of the GPH molecules adsorption at the surface of Fe (110) is presented. One may see that the inhibitors do not prefer any special places at the iron surface. The presented surface is homogenously covered by the isodensity shapes. It is especially seen for the protonated molecules. This homogeneous distribution is caused by the lack of atomic terraces and dense packing of atoms on the studied surface. The values of adsorption energy (*E*_ads_) for neutral form of studied molecules follow the order: GPH-3 (11.88 kcal mol^−1^) >GPH-2 (10.65 kcal mol^−1^) >GPH-1 (8.35 kcal mol^−1^), which is consisted with experimental order of inhibition efficiency. While for protonated and aqueous form of inhibitor molecules shows almost similar trends with slight variation.

## Conclusions

From the above studies on D-glucose derivatives of dihydropyrido [2,3-d:6,5-d′] dipyrimidine-2, 4, 6, 8(1H,3H, 5H,7H)-tetraone (GPHs) as corrosion inhibitors using gravimetric, electrochemical impedance spectroscopy (EIS), potentiodynamic polarization, scanning electron microscopy (SEM), energy dispersive X-ray spectroscopy (EDX), atomic force microscopy (AFM), quantum chemical calculations and Monte Carlo simulations techniques, it is concluded that:

(1) All studied molecules act as good inhibitors for mild steel corrosion in 1 M hydrochloric acid solution and the order of inhibition efficiencies for both gravimetric and electrochemical results is GPH-3 (97.82%) >GPH-2 (95.21%) >GPH-1(93.91%).The presence of electron releasing hydroxyl (-OH) and methoxy (-OCH_3_) groups in GPH-2 and GPH-3 respectively, showed the higher inhibition efficiency as compared to inhibitor without any substituents (GPH-1). Furthermore, the inhibitor molecule (GPH-3) having -OCH_3_ group exhibits the higher inhibition performance as compared to the inhibitor having -OH group.

(2) Adsorption of the inhibitor molecules on the metallic surface followed the Langmuir adsorption isotherm.

(3) Potentiodynamic polarization study revealed that studied inhibitor molecules acted as mixed type inhibitors but predominantly cathodic in nature.

(4) SEM, EDX and AFM analyses further confirmed the protecting ability of the inhibitor molecules.

(5) Quantum chemical calculations carried out for neutral, protonated and aqueous forms of the inhibitors supported the experimental order of inhibition efficiency. Values of E_HOMO_, electronegativity (*χ*) and dipole moment (*μ*) were consisted with experimental order of inhibition efficiency. Was also confirmed that inhibition increases in presence of electron releasing -OH and -OCH_3_ groups.

(6) Monte Carlo simulations study revealed that values of adsorption energy increased in presence of electron releasing -OH and -OCH_3_ groups. However, the values of *E*_ads_ did not showed any regular trend for aqueous and protonated inhibitor molecules.

(7) The results of weight loss, electrochemical, quantum chemical calculations and Monte Carlo were in good agreement.

## Additional Information

**How to cite this article:** Verma, C. *et al*. Corrosion inhibition of mild steel in 1 M HCl by D-glucose derivatives of dihydropyrido [2,3-d:6,5-d′] dipyrimidine-2, 4, 6, 8(1H,3H, 5H,7H)-tetraone. *Sci. Rep.* **7**, 44432; doi: 10.1038/srep44432 (2017).

**Publisher's note:** Springer Nature remains neutral with regard to jurisdictional claims in published maps and institutional affiliations.

## Supplementary Material

Supplementary Information File

## Figures and Tables

**Table 1:** Chemical structures and IUPAC names and abbreviation used for studied inhibitor molecules (GPHs).

Inhibitor 1	Inhibitor 2	Inhibitor 3
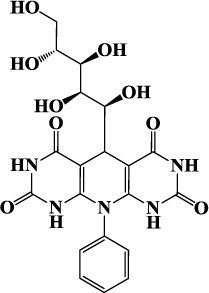	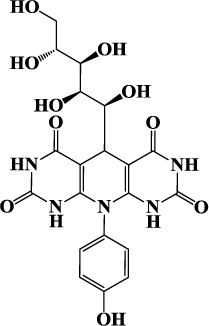	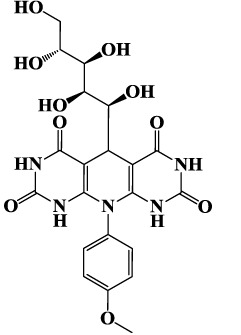
5-((1S,2R,3R,4R)-1,2, 3,4,5-pentahy droxy- pentyl)-10-phenyl-9,10-dihydropyrido[2,3-d:6,5-d′]dipyrimidine-2,4,6, 8 (1H,3H,5H, 7H)-tetraone	10-(4-hydroxyphenyl)-5-((1S,2R,3R,4R)-1,2, 3,4,5-pentahydroxy pentyl)-9,10-dihydro pyrido[2,3-d:6,5-d′] dipyrimidine-2,4,6,8 (1H,3H,5H,7H)-tetraone	10-(4-(methoxyphenyl)-5-((1S,2R,3R,4R)-1,2,3, 4,5-pentahydroxy pentyl) -9,10-dihydro pyrido[2,3-d:6,5-d′]dipyrimidine-2,4,6,8(1H,3H,5H,7H)-tetraone
**(GPH-1)**	**(GPH-2)**	**(GPH-3)**

**Table 2:** The weight loss parameters obtained for mild steel in 1 M HCl containing different concentrations of GPHs.

Inhibitors	Conc (mol/L)	Weight loss (mg)	*C*_R_ (mg cm^−2^ h^−1^)	Inhibition efficiency (*η* %)	Surface coverage (*θ*)
Blank	—	230	7.66	—	—
GPH-1	2.88 × 10^−5^	78	2.60	66.08	0.6608
	5.76 × 10^−5^	38	1.26	83.47	0.8347
	8.65 × 10^−5^	22	0.73	90.43	0.9043
	10.15 × 10^−5^	14	0.46	93.91	0.9391
GPH-2	2.88 × 10^−5^	63	2.10	72.60	0.7260
	5.76 × 10^−5^	29	0.96	87.39	0.8739
	8.65 × 10^−5^	16	0.53	93.04	0.9304
	10.15 × 10^−5^	11	0.36	95.21	0.9521
GPH-3	2.88 × 10^−5^	58	1.93	74.78	0.7478
	5.76 × 10^−5^	21	0.70	90.86	0.9086
	8.65 × 10^−5^	10	0.33	95.65	0.9565
	10.15 × 10^−5^	5	0.16	97.82	0.9782

**Table 3:** Variation of *C*_R_ and *η* % with temperature in absence and presence of optimum concentration of GPHs in 1 M HCl.

Temperature (K)	Corrosion rate (*C*_R_) (mg cm^−2^ h^−1^) and Inhibition efficiency (*η%*)							
	Blank		GPH-1		GPH-2		GPH-3	
	*C*_R_	*η*%	*C*_R_	*η*%	*C*_R_	*η*%	*C*_R_	*η*%
308	7.66	—	0.46	93.91	0.36	95.21	0.16	97.82
318	11.0	—	1.20	89.09	0.96	91.21	0.56	94.84
328	14.3	—	2.66	81.39	2.30	83.95	1.60	88.83
338	18.6	—	5.43	70.89	4.96	73.39	4.13	77.85

**Table 4:** Values of *K*_ads_ and 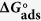 for mild steel in absence and presence of optimum concentration of GPHs in 1 M HCl at different studied temperature.

Inhibitor	*K*_ads_ (10^4^M^−1^)				− 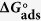 (k Jmol^−1^)			
Temperature	308	318	328	338	308	318	328	338
GPH-1	1.85	0.98	0.52	0.29	35.45	34.92	34.32	33.72
GPH-2	2.38	1.24	0.62	0.33	36.10	35.56	34.81	34.07
GPH-3	5.38	2.20	0.95	0.42	38.19	37.07	35.95	34.75

**Table 5:** Tafel polarization parameters for mild steel in 1M HCl solution in absence and presence of different concentrations of GPHs.

Inhibitor	*Conc (mol/L*)	*E*_*corr*_ (*mV/SCE*)	*β*_*a*_(*mV/dec*)	*β*_*c*_(*mV/dec*)	*i*_*corr*_ (*μA/cm*^2^)	*η%*
Blank	—	−445	70.5	114.6	1150	—
GPH-1	2.88 × 10^−5^	−530	67.3	159.8	432.0	62.43
	5.76 × 10^−5^	−506	64.2	183.3	189.0	83.56
	8.65 × 10^−5^	−555	64.2	90.50	103.0	91.04
	10.15 × 10^−5^	−504	86.7	89.80	72.0	93.73
GPH-2	2.88 × 10^−5^	−534	65.5	161.1	344.0	70.08
	5.76 × 10^−5^	−481	66.4	139.7	178.0	84.52
	8.65 × 10^−5^	−508	67.0	125.1	98.0	91.47
	10.15 × 10^−5^	−461	59.9	129.9	67.9	94.09
GPH-3	2.88 × 10^−5^	−489	67.5	132.8	298.0	74.08
	5.76 × 10^−5^	−489	81.6	107.8	132.0	88.52
	8.65 × 10^−5^	−554	93.5	153.8	83.0	92.78
	10.15 × 10^−5^	−478	63.8	166.4	32.30	97.19

**Table 6:** EIS parameters obtained for mild steel in 1 M HCl in absence and presence of different concentrations of GPHs.

Inhibitor	*Conc (mol/L*)	*R*_*s*_ (*Ω cm*^2^)	*R*_*ct*_ (*Ω cm*^2^)	*n*	*C*_*dl*_ (*μF cm*^*−2*^)	*η%*
Blank	—	1.12	9.58	0.827	106.21	—
GPH-1	2.88 × 10^−5^	1.10	25.45	0.905	78.70	62.35
	5.76 × 10^−5^	1.25	65.73	0.881	77.67	85.42
	8.65 × 10^−5^	1.16	111.03	0.843	58.84	91.37
	10.15 × 10^−5^	1.41	170.38	0.835	55.74	94.37
GPH-2	2.88 × 10^−5^	0.936	34.27	0.895	59.58	72.04
	5.76 × 10^−5^	0.785	69.77	0.815	57.09	86.26
	8.65 × 10^−5^	0.89	127.01	0.846	56.27	92.45
	10.15 × 10^−5^	1.085	176.91	0.821	40.62	94.58
GPH-3	2.88 × 10^−5^	1.13	39.05	0.871	57.72	75.46
	5.76 × 10^−5^	0.74	75.15	0.853	55.00	87.25
	8.65 × 10^−5^	1.29	135.40	0.829	47.36	92.92
	10.15 × 10^−5^	1.67	265.72	0.845	37.41	96.39

**Table 7:** Electronic parameters calculated for GPHs neutral and protonated molecules in vacuum and water using DFT/B3LYP method.

Parameters	GPH-1	GPH-2	GPH-3
Molecule in vacuum			
*E*_HOMO_ [eV]	−6.31	−6.28	−6.24
*E*_LUMO_ [eV]	−1.83	−1.80	−1.74
Δ*E*_LUMO-HOMO_ [eV]	4.48	4.48	4.50
*μ* [D]	6.82	7.25	8.17
*χ*	4.07	4.04	3.99
Protonated molecules			
*E*_HOMO_ [eV]	−10.02	−9.98	−9.89
*E*_LUMO_ [eV]	−5.50	−5.45	−5.39
Δ*E*_LUMO-HOMO_ [eV]	4.52	4.53	4.50
*μ*[D]	5.29	5.08	5.29
*χ*	7.76	7.72	7.64
Molecule in water			
*E*_HOMO_ [eV]	−6.26	−6.27	−6.26
*E*_LUMO_ [eV]	−1.76	−1.75	−1.74
Δ*E*_LUMO-HOMO_ [eV]	4.50	4.52	4.52
*μ*[D]	11.07	11.10	12.30
*χ*	4.01	4.01	4.00

**Table 8:** Average adsorption energy of the GPH molecules at the Fe (110) surface and the average total energy of the investigated systems.

Systems	Total Energy (kcal mol^−1^)	Adsorption Energy (kcal mol^−1^)
neutral molecule		
Fe (110) + GPH-1	79.48	−8.35
Fe (110) + GPH-2	71.64	−10.65
Fe (110) + GPH-3	73.30	−11.88
protonated molecule		
Fe (110) + GPH-1	163.24	−47.46
Fe (110) + GPH-2	153.74	−45.20
Fe (110) + GPH-3	153.51	−43.19
molecule in water		
Fe (110) + GPH-1	36.10	−33.29
Fe (110) + GPH-2	24.25	−38.02
Fe (110) + GPH-3	33.32	−31.25
