# Supplementary material for: Corrosion inhibition of mild steel in 1M HCl by D-glucose derivatives of dihydropyrido [2,3-d:6,5-d′] dipyrimidine-2, 4, 6, 8(1H,3H, 5H,7H)-tetraone
Source: Sci Rep. 2017 Mar 20;7:44432. doi: 10.1038/srep44432 (PMC5357850; doi:10.1038/srep44432)
Supplement: Supplementary Information File [file srep44432-s1.pdf]

SUPPORTING INFORMATION

Corrosion inhibition of mild steel in 1 M HCl by D-glucose derivatives of dihydropyrido [2,3-d:6,5-d'] dipyrimidine-2, 4, 6, 8(1H,3H, 5H,7H)-tetraone

Chandrabhan Verma[1], M. A. Quraishi[1,*], K. Kluza[2], M. Makowska-Janusik[2], Lukman O. Olasunkanmi[3,4], Eno E. Ebenso[3,*]

[1]Department of Chemistry, Indian Institute of Technology, Banaras Hindu University, Varanasi 221005, India
[2] Institute of Physics, Faculty of Mathematics and Natural Science, Jan Dlugosz University, Al. Armii Krajowej 13/15, 42-200 Czestochowa, Poland
[3] Material Science Innovation & Modelling (MaSIM) Research Focus Area, Faculty of Agriculture, Science and Technology, North-West University (Mafikeng Campus), Private Bag X2046, Mmabatho 2735, South Africa
[4] Department of Chemistry, Obafemi Awolowo University, Ile-Ife 220005, Nigeria

***Corresponding author: Ph.no. +91-9307025126; Fax: +91- 542- 2368428**
E-mail: maquraishi.apc@itbhu.ac.in; maquraishi@rediffmail.com; Eno.Ebenso@nwu.ac.za

5-((1S,2R,3R,4R)-1,2, 3,4,5-pentahy droxy- pentyl)-10-phenyl-9,10-dihydropyrido[2,3-d:6,5-d']dipyrimidine-2,4,6, 8 (1H,3H,5H,7H)-tetraone **(GPH-1):**

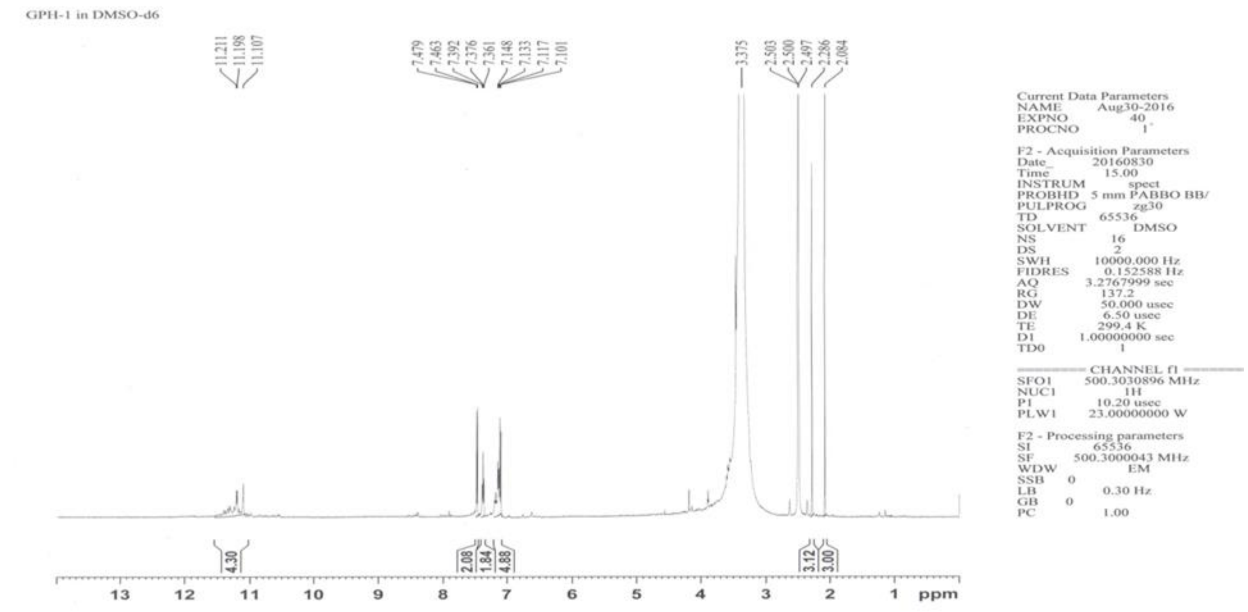

1H NMR

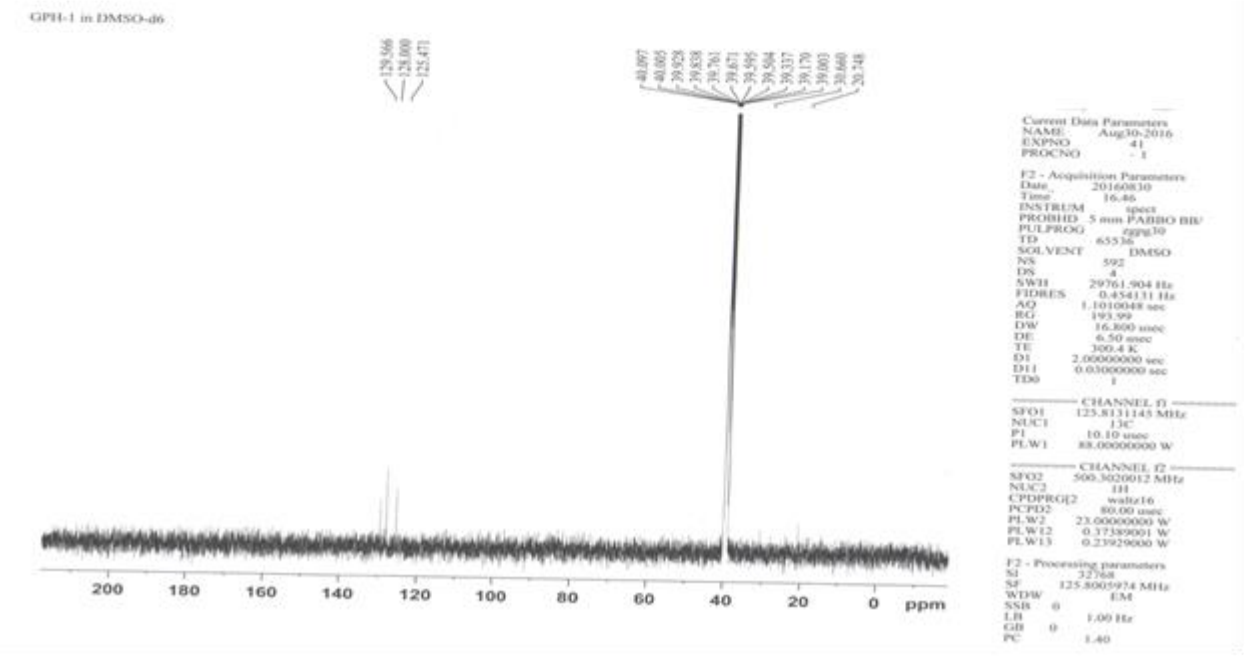

13C NMR

10-(4-hydroxyphenyl)-5-((1S,2R,3R,4R)-1,2, 3,4,5-pentahydroxy pentyl)-9,10-dihydro pyrido[2,3-d:6,5-d'] dipyrimidine-2,4,6,8 (1H,3H,5H,7H)-tetraone **(GPH-2)**

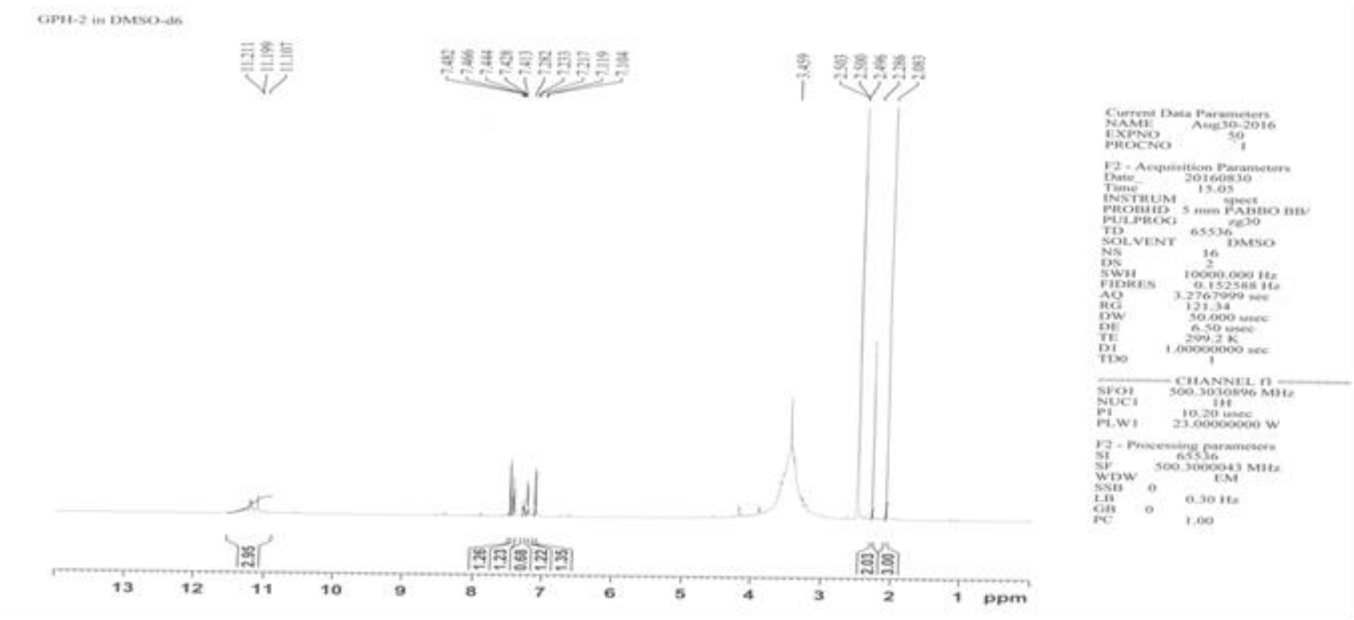

1H NMR

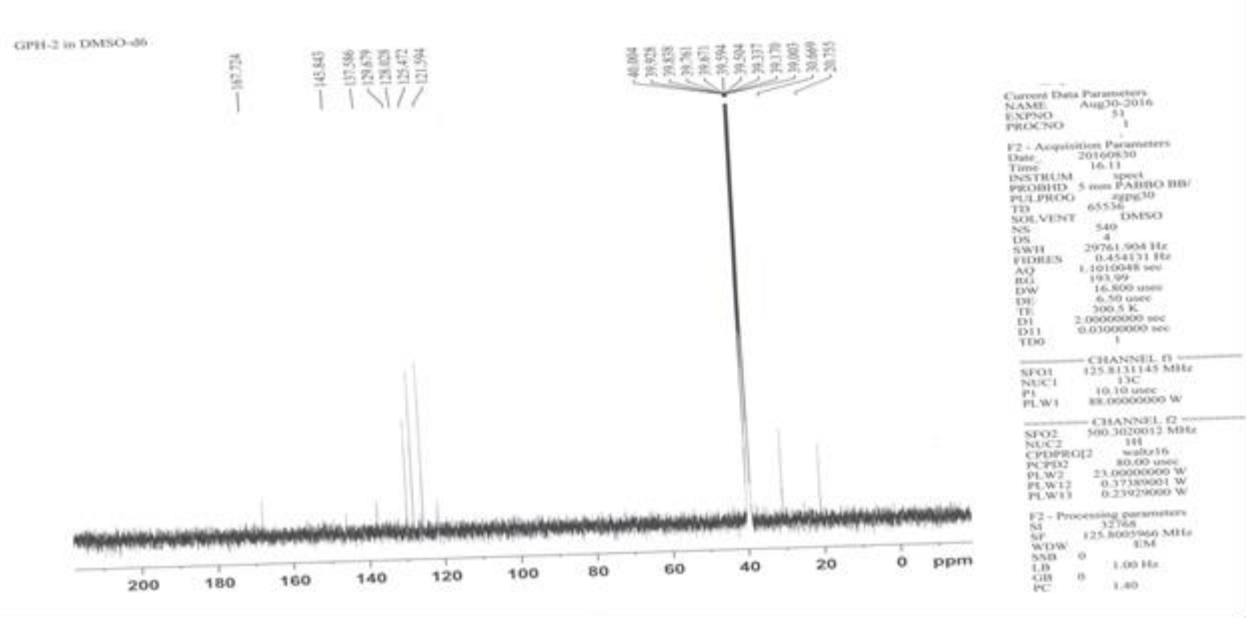

13C NMR

10-(4-(methoxyphenyl)-5-((1S,2R,3R,4R)-1,2,3, 4,5-pentahydroxy pentyl) -9,10-dihydro pyrido[2,3-d:6,5-d']dipyrimidine-2,4,6,8(1H,3H,5H,7H)-tetraone **(GPH-3)**

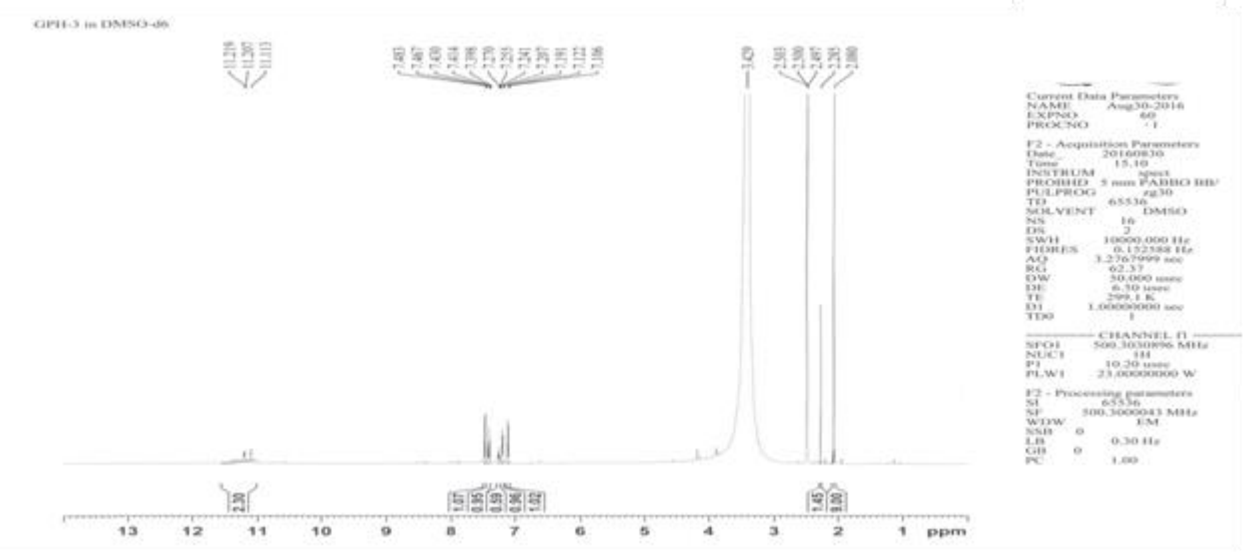

1H NMR

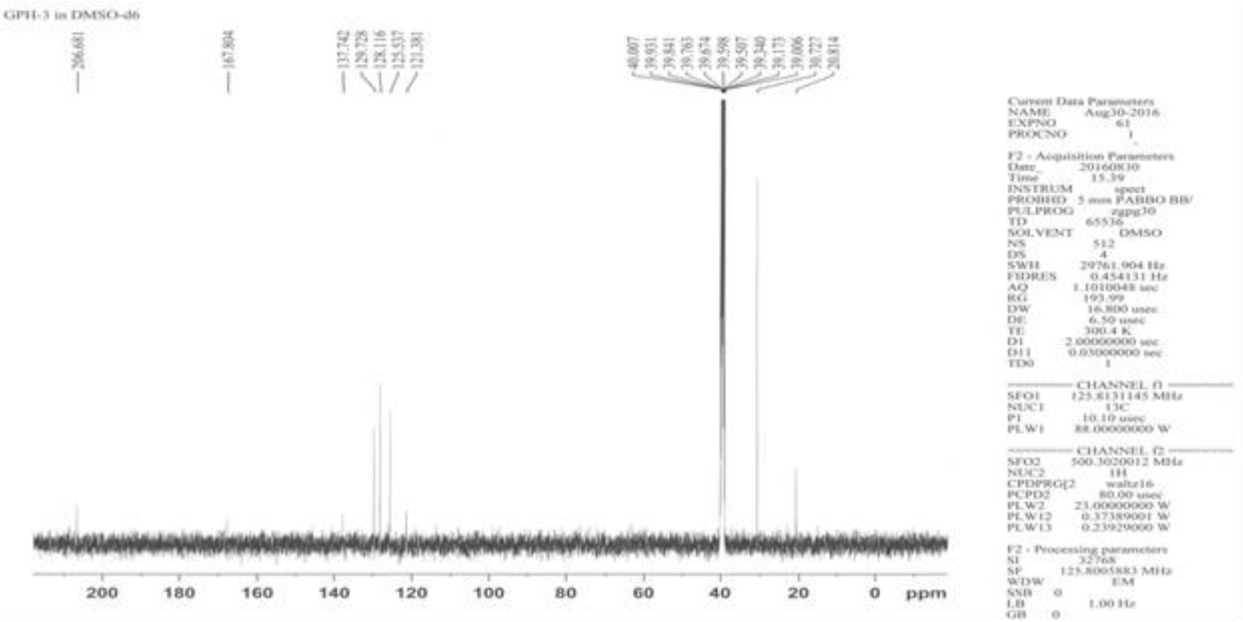

13C NMR

Fig. S1: 1H and 13C NMR spectra of synthesized compounds

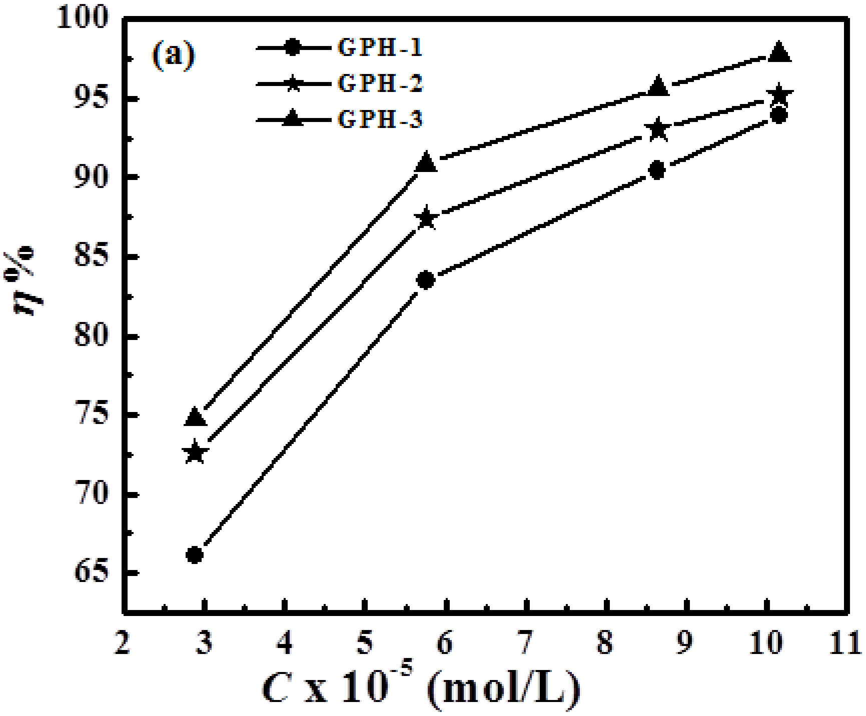

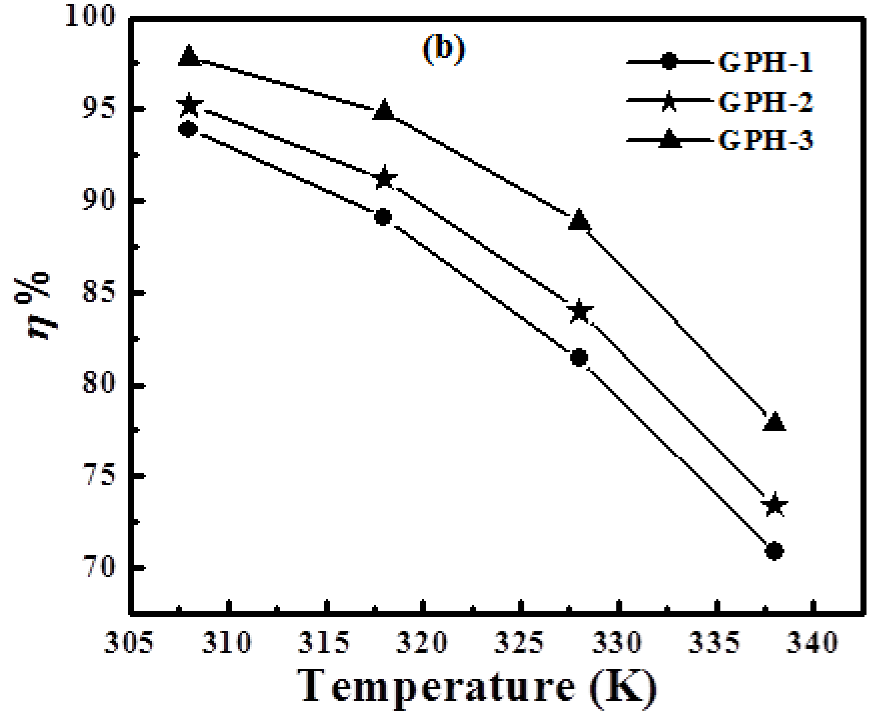

Fig. S2a-b: (a): Variation of inhibition efficiency with inhibitors concentrations

(b) Variation of inhibition efficiency with solution temperatures

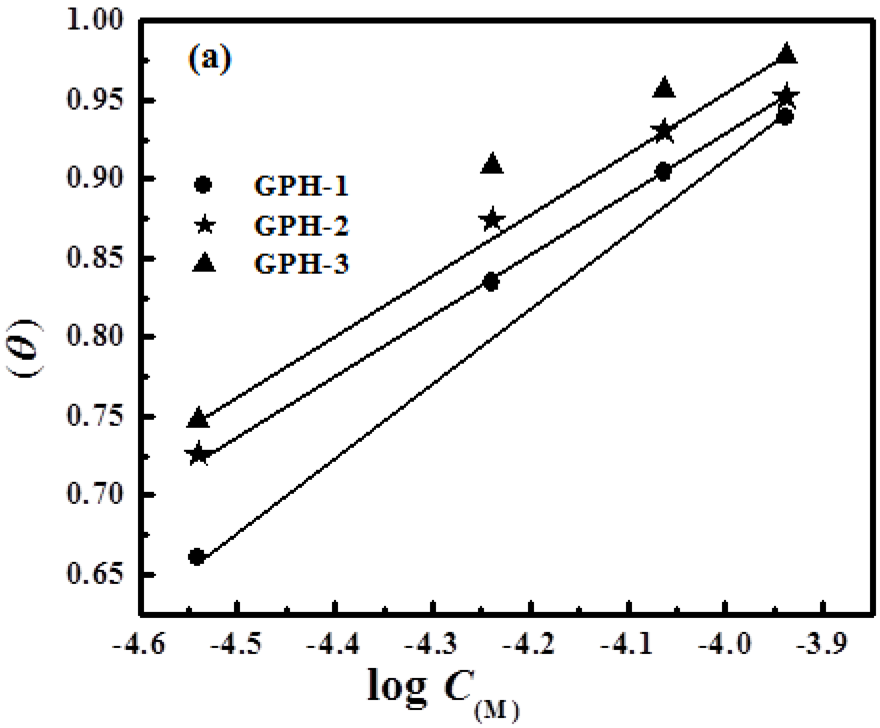

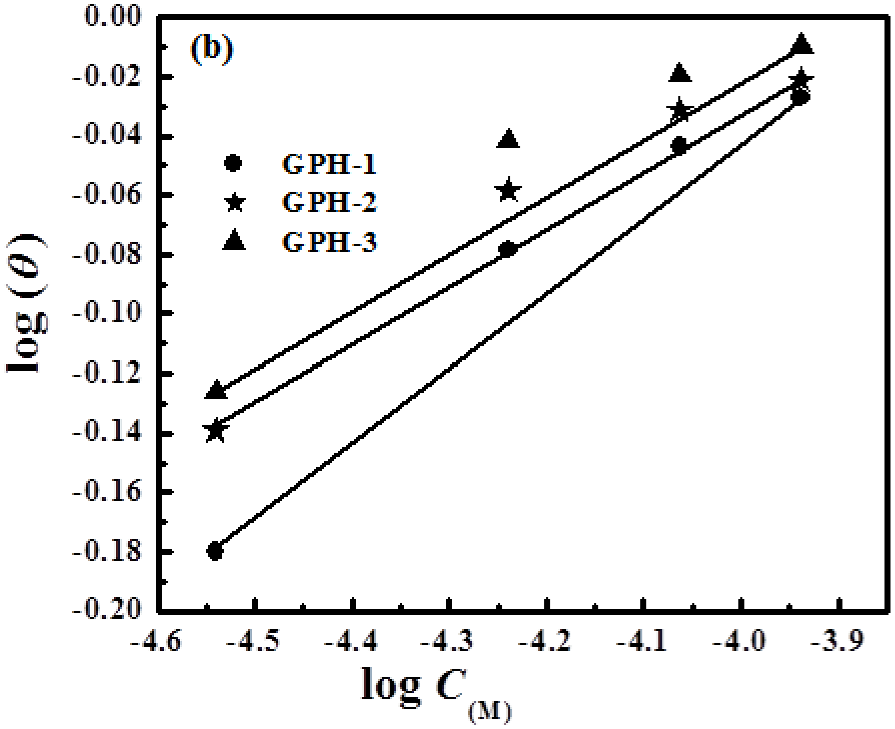

Fig. S3: (a) Temkin and (b) Freundluich adsorption isotherm plots for the corrosion of mild steel in 1 M HCl without and with the inhibitors.

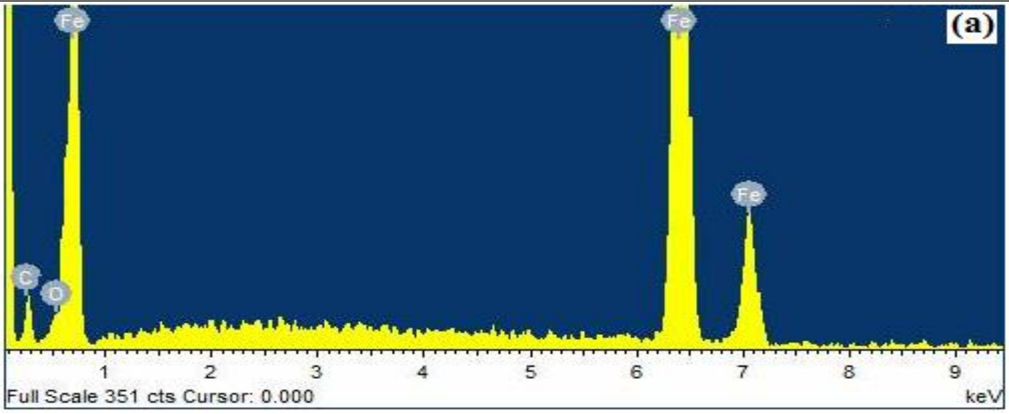

Element	Wt%	At%
C	07.18	34.94
O	02.18	2.27
Fe	89.39	62.77
Matrix	Correction	ZAF

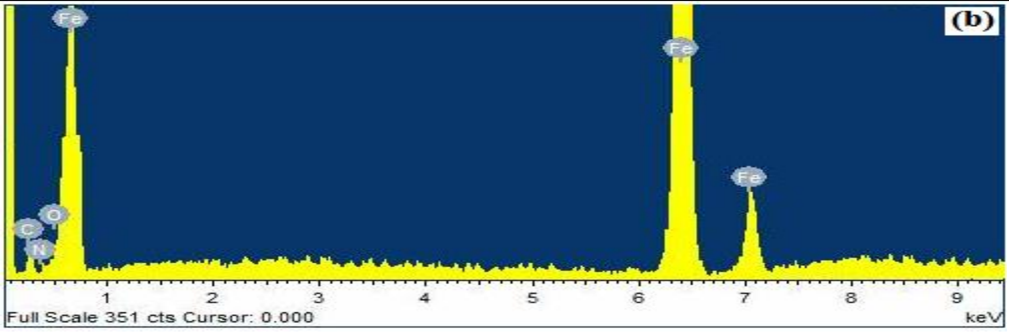

Element	Wt%	At%
C	08.27	23.84
N	02.96	02.13
O	04.32	04.46
Fe	84.24	69.23
Matrix	Correction	ZAF

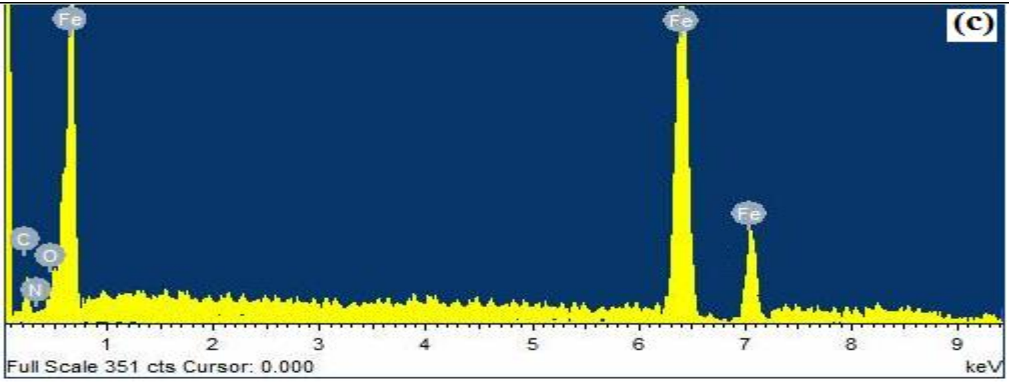

Element	Wt%	At%
C	06.28	26.13
N	02.17	05.22
O	04.87	7.56
Fe	85.98	60.43
Matrix	Correction	ZAF

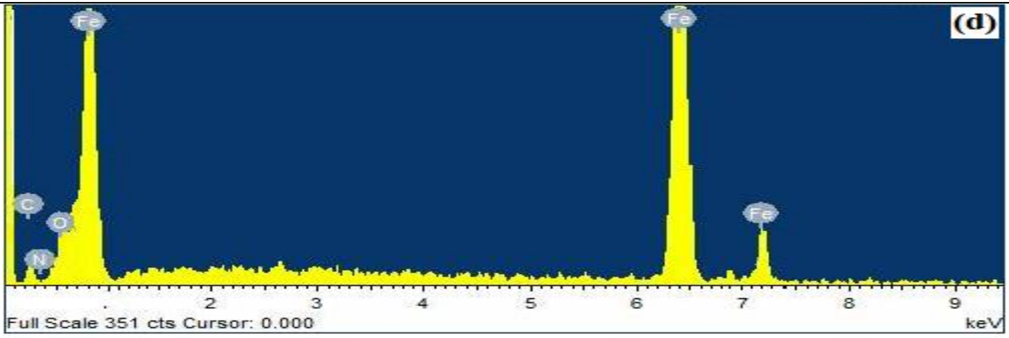

Element	Wt%	At%
C	06.56	024.72
N	02.34	04.52
O	05.42	08.54
Fe	84.96	63.13
Matrix	Correction	ZAF

Figure S4: EDX spectra and corresponding elemental

Table S1: Slopes, intercepts, and regression coefficients (R^2) for Langmuir and Temkin adsorption isotherms plots for investigated inhibitors

**Inhibitor**	**Langmuir adsorption isotherm**			**Temkin adsorption isotherm**			**Freundluich isotherm**		
	slope	intercept	(R^2)	slope	intercept	(R^2)	slope	intercept	(R^2)
**GPH-1**	1.4831	7.0108	0.9978	0.4696	2.8047	0.9806	0.258	1.0000	0.9678
**GPH-2**	1.4639	7.0636	0.9990	0.3843	2.4829	0.9700	0.2002	0.7774	0.9594
**GPH-3**	1.9318	9.2202	0.9947	0.3897	2.5328	0.9549	0.1974	0.7791	0.9430

Table S2: Values of slope, intercept, correlation coefficient and phase angle calculated form Bode plots of the in absence and presence of studied compounds

inhibitors	Slope Decades/Decade	Intercept (kohm)	Correlation coefficient (R^2)	Phase angle
**Blank**	-0.4805	0.0926	-0.99841	-41.3
**GPH-1**	-0.7193	0.4389	0.99941	-58.6
	-0.7507	0.8091	-0.99976	-61.9
	-0.7739	0.2002	-0.99984	-68.4
	-0.7838	0.1095	-0.9998	-69.5
**GPH-2**	-0.7721	0.7736	-0.99908	-64.3
	-0.7434	0.8921	-0.99960	-64.5
	-0.7664	1.1804	0.99989	-65.7
	-0.7921	1.6581	-0.99978	-68.6
**GPH-3**	-0.6775	0.7774	-0.99827	-58.9
	-0.7668	0.5279	0.99980	-63.4
	-0.7760	0.5590	0.99945	-66.2
	-0.7713	1.1243	0.99941	-67.8
